# Predicting tissue specific cis-regulatory modules in the human genome using pairs of co-occurring motifs

**DOI:** 10.1186/1471-2105-13-25

**Published:** 2012-02-07

**Authors:** Hani Z Girgis, Ivan Ovcharenko

**Affiliations:** 1 Computational Biology Branch, National Center for Biotechnology Information, National Library of Medicine, National Institutes of Health 9600 Rockville Pike, Bethesda, MD 20896, USA

## Abstract

**Background:**

Researchers seeking to unlock the genetic basis of human physiology and diseases have been studying gene transcription regulation. The temporal and spatial patterns of gene expression are controlled by mainly non-coding elements known as cis-regulatory modules (CRMs) and epigenetic factors. CRMs modulating related genes share the regulatory signature which consists of transcription factor (TF) binding sites (TFBSs). Identifying such CRMs is a challenging problem due to the prohibitive number of sequence sets that need to be analyzed.

**Results:**

We formulated the challenge as a supervised classification problem even though experimentally validated CRMs were not required. Our efforts resulted in a software system named CrmMiner. The system mines for CRMs in the vicinity of related genes. CrmMiner requires two sets of sequences: a mixed set and a control set. Sequences in the vicinity of the related genes comprise the mixed set, whereas the control set includes random genomic sequences. CrmMiner assumes that a large percentage of the mixed set is made of background sequences that do not include CRMs. The system identifies pairs of closely located motifs representing vertebrate TFBSs that are enriched in the training mixed set consisting of 50% of the gene loci. In addition, CrmMiner selects a group of the enriched pairs to represent the tissue-specific regulatory signature. The mixed and the control sets are searched for candidate sequences that include any of the selected pairs. Next, an optimal Bayesian classifier is used to distinguish candidates found in the mixed set from their control counterparts. Our study proposes 62 tissue-specific regulatory signatures and putative CRMs for different human tissues and cell types. These signatures consist of assortments of ubiquitously expressed TFs and tissue-specific TFs. Under controlled settings, CrmMiner identified known CRMs in noisy sets up to 1:25 signal-to-noise ratio. CrmMiner was 21-75% more precise than a related CRM predictor. The sensitivity of the system to locate known human heart enhancers reached up to 83%. CrmMiner precision reached 82% while mining for CRMs specific to the human CD4^+ ^T cells. On several data sets, the system achieved 99% specificity.

**Conclusion:**

These results suggest that CrmMiner predictions are accurate and likely to be tissue-specific CRMs. We expect that the predicted tissue-specific CRMs and the regulatory signatures broaden our knowledge of gene transcription regulation.

## Background

Understanding gene regulation is crucial to understand human development and to uncover the genetic basis of physiological and pathological processes. DNA sequences known as cis-regulatory modules (CRMs) play an important role in the tempo-spatial regulation of a gene. CRMs may be located a few dozen nucleotides from the transcription start sites or millions of nucleotides way. To activate a gene in a specific tissue, transcription factors (TFs) bind to their binding sites (TFBSs) in a CRM. Often, a co-activator protein binds to the TF complex and to RNA Polymerase II, bringing the CRM into close proximity to the promoter to start transcription [1].

Experimental methods to identify tissue-specific CRMs are available. One method relies on detecting DNase I Hypersensitive regions that are strong makers of CRMs [2]. CRMs can also be recognized by identifying specific histone marks, such as H3K4me1, H3K4me3, or K3K27ac, for example [3]. Visel et al. [4] and Blow et al. [5] used chromatin immunoprecipitation followed by sequencing (ChIP-seq) to detect enhancers. The antibodies used in the ChIP-seq method target the co-activator P300 protein that forms a complex with TFs binding to a CRM. Candidate sequences identified by the above methods are usually fused to a reporter gene to be tested in transgenic animals. Although experimental methods are effective in identifying tissue specific enhancers, they are relatively expensive, time consuming, and may require animal models. In addition, experimental methods are not perfect. For example, 87% and 62% of the P300 peaks obtained in [4] and [5] showed the expected tissue-specific activity in transgenic mice.

Predicting CRMs from DNA sequences is a challenging problem in computational biology. Few computational methods take advantage of the availability of experimentally confirmed CRMs [6-9]. These methods apply supervised-learning algorithms to identify sequence features that are specific to the confirmed CRMs. Sequence features may include motifs representing TFBSs and words of specific length. Even though these methods are useful in identifying tissue specific CRMs, they cannot be applied when there are not enough known CRMs for a desired cell type. Since experimentally confirmed CRMs are not available for all tissues, alternative methods have been developed. Several of these methods depend on detecting clusters of predetermined TFBSs in a sequence [10-13]. These methods can be useful to search for a specific combination of TFBSs. However, they cannot be applied when the regulating TFs or their motifs are unknown. Searching for clusters of the same TFBS has been the underlying principle of related computational methods [14,15]. These methods can identify CRMs that include several instances of the same TFBS. They cannot identify those that include different TFBSs.

CRMs modulating co-expressed or co-regulated genes are likely to share TFBSs that comprise the regulatory signature. The main challenge facing the discovery of the regulatory signature of a group of related genes is the prohibitive number of all possible sets. To illustrate the difficulty, consider a set of 100 co-expressed genes. Each gene locus has 10 candidate sequences (this number can be much larger in reality). If only one sequence includes a cis-regulatory module of a gene, the total number of sets to be analyzed is 10^100^. Next, we illustrate how computational methods have attempted to circumvent this challenge.

Initially, computational methods limited the search for CRMs to the promoter regions or to the conserved elements within a few thousand nucleotides upstream of the transcription start sites (TSSs) [16-24]. Grad et al. [25] reduced the number of sequences to be analysed by searching for "similar short, conserved sequences" near the co-expressed genes. The Enhancer Identification (EI) method [26] searches for modules of TFBSs enriched in conserved sequences in the promoter regions or in the "three most conserved" sequences in the vicinity of tissue-specific genes. Further, the EI method reduces the number of sequences to be analysed by dynamically considering only sequences that include a tissue-specific TFBS module. Pairs of nearby co-occurring words or TFBSs can describe the regulatory signature of the promoters of tissues-specific genes [27-29]. The CRM-PI [30] method predicts CRMs based on pairs of interacting TFs by analysing "conserved regions" within 1.5 kbp upstream of the TSSs. Then, it scans the regions 5 kbp upstream of TSSs for segments that are enriched with the interacting TFs.

The above mentioned methods are successful in detecting tissue-specific CRMs modulating co-expressed or co-regulated genes. Nevertheless, their search scope is limited. CRMs can be located hundreds of thousands of nucleotides from their target genes [31,32]. Therefore, computational methods that can mine for tissue-specific CRMs among a large number of sequences are needed.

We designed and developed a probabilistic discriminative system, CrmMiner. CrmMiner attempts to overcome some of the limitations of the current state-of-the-art. Specifically, CrmMiner is designed to mine for CRMs in a large number of sequences. Hence, it has the potential to find CRMs thousands of nucleotides away from their target TSSs. Two principles provide the foundation for CrmMiner. First, CRMs, which regulate a group of tissue-specific genes, share TFBSs. Second, proteins never act in isolation. Therefore, pairs of motifs representing co-occurring TFBSs can be used to describe the tissue-specific regulatory signature.

CrmMiner overcomes the problem of mining for CRMs among a large number of background sequences by filtering out sequences that are not likely to include CRMs. The filtering step reduces the search space from thousands to a few hundreds of sequences. For example, the vicinity of a group of co-expressed genes includes thousands of sequences. A few hundreds of these sequences include CRMs. To reduce the number of sequences to be analyzed, during training CrmMiner identifies a subset of tens or a few hundreds of motif pairs that are enriched in this group of loci. At the same time, the system selects candidate sequences that include any of the selected pairs. This step results in set of a few hundred of sequences which are likely to include CRMs. Often, there are thousands of motif pairs that are enriched in a group of loci. However, selecting the candidate sequences based on the entire set of the enriched pairs would result in thousands of sequences. The majority of these sequences do not include CRMs. As a result, the filtering step would be ineffective. Therefore, we designed a greedy algorithm to select the final enriched pairs during training.

From the technical point of view, we formulated the task as a supervised classification problem. However, CrmMiner does not require experimentally validated CRMs. In sum, the contributions of our study are:

• The CrmMiner software,

• Putative CRMs specific to 62 human tissues and cell types, and

• Predicted regulatory signatures specific to 62 human tissues and cell types.

Next, we describe the classification algorithm and discuss the performance of CrmMiner.

## Methods

### CrmMiner

In this section, we present our discriminative probabilistic system, CrmMiner. The goal of CrmMiner is mining for CRMs in the vicinity of a group of co-expressed or functionally related genes.

#### Input

The input to CrmMiner is two sets of sequences: a mixed set and a control set. The mixed set is assumed to include cis-regulatory modules (CRMs) mixed with a large number of background sequences. In our experiments, the mixed sets consisted of sequences found in the vicinity of related genes. The control set consists of background sequences that are unlikely to include CRMs. A well-known problem in the field of machine learning is over-fitting the training data. Over-fitting is manifested by an excellent performance on the training data; however, the performance on new testing data is poor. In other words, the algorithm does not perform as well on the testing data. To guard against over-fitting, the input sequences are partitioned into three sets: (1) training set, sequences in 50% of the loci; (2) validation set, sequences in 25% of the loci; and (3) testing set, sequences of 25% of the loci. We constructed a control set for each of the three mixed sets.

#### Output

The output of CrmMiner is the predicted CRMs and their regulatory signature. The signature is represented by pairs of motifs of transcription factor binding sites (TFBSs). Those CRMs should share the signature completely or partially.

#### Overview

**Figure 1:**
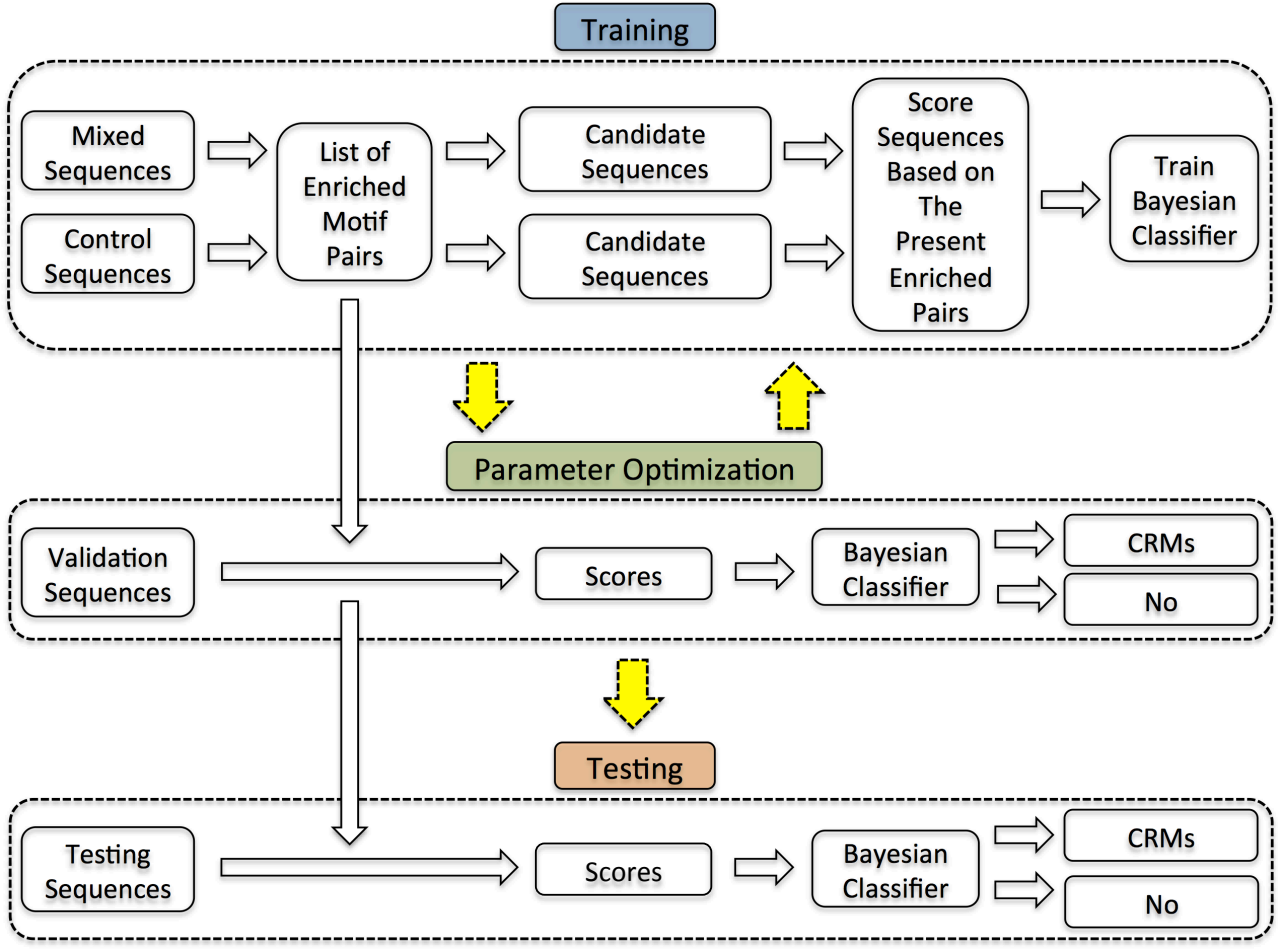
**Method overview**. The diagram illustrates the workflow of the system. During training, the system contrasts sequences from the mixed set to control sequences to identify motif pairs that are enriched in the mixed set. The system identifies and scores sequences that include at least one of the enriched pairs. A Bayesian classifier is trained on the scores to distinguish candidate sequences in the mixed set from candidates in the control set. During validation, the list of pairs and the trained classifier are used to classify sequences in the validation set. The training and the validation are repeated to find the parameters that result in the best performance on the validation set. Finally, CrmMiner is tested on sequences in the testing set.

Our procedure to predict CRMs consists of the following steps (Figure 1):

• Scan sequences for motifs of known vertebrate TFBSs.

• Train CrmMiner on the training set:

**- **Identify pairs of motifs that meet specific criteria.

**- **Search the mixed and control sets for candidate sequences that include at least one of the selected pairs.

**- **Score candidate sequences.

**- **Determine a threshold that separates the scores of candidates found in the mixed set from those found among the controls.

**- **Predict candidate sequences found in the mixed set as CRMs if their scores are above the threshold.

• Validate CrmMiner's performance on the validation set. Predict CRMs according to the list of pairs and the threshold, both of which are determined during training.

• Repeat training and validation to find the parameters that result in the best performance on the validation set.

• Test CrmMiner with the parameters determined in the previous step on the testing set.

#### Scanning sequences for motifs of known vertebrate TFBSs

We obtained 966 position weight matrices representing motifs of binding sites of known vertebrate transcription factors from the TRANSFAC (release 2010.4) database [33]. The MAST program [34] scanned each sequence and its reverse complement to detect the presence of the 966 motifs. In the case where multiple motifs overlapped, all of them were kept.

#### Selecting a set of motif pairs enriched in the training mixed set

The initial set of motif pairs is selected according to three criteria. First, the two non-overlapping motifs have to be near one another and separated by no more than a certain number of nucleotides (system parameter). Second, a pair has to occur at least a certain number of times in the mixed set (system parameter). For example, suppose that the mixed set is expected to include 100 CRMs. The user may require a pair of motifs to be present in at least 10% of the CRMs. In this case, CrmMiner considers pairs that occur at least 10 times in the mixed set. Third, the pair is more enriched in the mixed set than the control set. The enrichment of a pair *p *is measured by the E-value_p_ which is calculated as follows:

(1) $$\text{E-valu}{\text{e}}_{p}=\frac{l_{c}}{l_{m}}\times \frac{n_{m}}{n_{c}}$$

*l_c _*and *l_m _*are the sum of sequence lengths in the control and mixed sets, and *n_m _*and *n_c _*are the number of times a pair occurred in the mixed and control sets. Pairs that are not enriched in the mixed set (i.e. E-value ≤ 1) are excluded. However, not all enriched pairs are considered. The E-value of a pair has to be above the mean E-value of the enriched pairs, and less than 2-5 standard deviations above the mean (system parameter). We avoid selecting pairs that are extremely enriched in the training set because they may not be enriched in other sets. In other words, those pairs may be potential outliers. The three parameters, discussed in this step, are adjusted automatically during validation.

#### Selecting candidate sequences

The goal of this step is to select a set of sequences that are more enriched with sequences found in the mixed set than with controls. To this end, CrmMiner uses a greedy algorithm to collect such a set. For a set *h*, we define the E-value*_h _*to measure the enrichment of *h *with sequences from the mixed set as follows:

(2) $$\text{E-valu}{\text{e}}_{h}=\frac{l_{c}}{l_{m}}\times \frac{r_{m}}{r_{c}}$$

*l_c _*and *l_m _*are defined as before, and *r_m _*and *r_c _*are the number of sequences from the mixed and control sets in *h*. The algorithm depends on the enriched pairs determined in the previous step. The algorithm begins by sorting the enriched pairs in descending order. It considers the top *k *(system parameter) enriched pairs in the list. For each one of the *k *pairs, CrmMiner calculates the enrichment value that may be obtained if sequences including this pair would be added to the set of candidate sequences (initially empty). CrmMiner selects the pair that results in the best E-value*_h_*. The sequences, which include this pair, are added to the set of candidate sequences. The chosen motif pair is removed from the sorted list and added to a list representing the regulatory signature. Then, the algorithm repeats the search through the top *k *pairs in the updated sorted list. The rational for this step is that, although a pair could have the highest E-value*_p_*, it may not result in the best E-value*_h_*. Therefore, examining other highly enriched pairs should provide a better alternative. The algorithm stops if at least one of the following three criteria is violated:

• The enrichment of the candidate sequences with sequences from the mixed set is above a threshold (system parameter);

• The number of sequences from the mixed set among the candidate sequences is less than the maximum allowed (system parameter); and

• The number of pairs, representing the regulatory signature, is less than the maximum number of candidate sequences gathered from the mixed set (system parameter).

The algorithm selects a list of motif pairs representing the regulatory signature. In addition, it outputs candidate sequences found in the mixed and control sets. Based on experimental results, it is recommended to set the minimum E-value*_h _*to 3-5 fold, the maximum number of candidate sequences collected from the mixed set to 1-5 times the number of the expected CRMs, and *k *to 1-10. We determine the values of these parameters automatically, by trying several combinations on the validation set.

To illustrate how the algorithm works, suppose that the training set includes 80 gene loci, where one CRM is expected to regulate each gene. The user may wish to set *k *to 3, the minimum E-value*_h _*to 3 fold, and the maximum number of candidate sequences collected from the mixed set to 240 (3 × 80). Using 240 allows, initially, at most one true positive in every three sequences because the user assumed that there are 80 CRMs. Some of these sequences will be removed in the next stage. Let *P *= {*p*_1_, *p*_2_, *p*_3_, *p*_4_, ..., *p_n_*} be the sorted list of motif pairs such that ${\text{E-value}}_{p_{i}}\geq {\text{E-value}}_{p_{j}}$, ∀*i *<*j*. Let *S *= {} be the set of motif pairs representing the regulatory signature. Let *H *= {} be the set of candidate sequences. Notice that *H *can include sequences from the mixed and control sets. The algorithm considers adding *p*_1_, *p*_2_, or *p*_3 _to *S*. Suppose that motif pair *p*_1 _results in the highest set enrichment, E-value*_h_*. Then, *p*_1 _is removed from *P *and added to *S*. Sequences including this pair are added to *H*. Then, the algorithm considers pairs *p*_2_, *p*_3_, or *p*_4_. CrmMiner stops if (1) the E-value*_h _*drops below 3 fold, (2) *H *includes more than 240 sequences from the mixed set, or (3) the number of the selected pairs, |*S*|, is more than 240.

#### Predicting CRMs

CrmMiner searches the mixed and control sets for sequences that include at least one of the final motif pairs. We refer to this set of pairs as the tissue-specific regulatory signature. Many of these pairs are expected to occur in control sequences by chance. To distinguish between candidates found in the mixed set (potential CRMs) and those found among controls, CrmMiner trains a Bayesian classifier [35]. A sequence is scored as the sum of the E-values*_p _*of the signature motif pairs present in it. The goal is to find a score above which a sequence is more likely to be from the mixed set than from the control set. Our assumption is that a candidate sequence found in the mixed set should include more than one of the enriched pairs. In addition, the majority of control sequences should include at most one of the enriched pairs. Therefore, the scores of candidate sequences gathered from the mixed set should be higher than those of their control counterparts. One way to find such a threshold score is to calculate the posterior probabilities of a sequence being from the mixed set or from the control set, given its score is greater than or equal to the threshold (Equations 3 and 4).

(3) $$p\left(C=c_{1}|T\geq t\right)=\frac{p\left(C=c_{1}\right)\times p\left(T\geq t|C=c_{1}\right)}{p\left(T\geq t\right)}$$

(4) $$p\left(C=c_{2}|T\geq t\right)=\frac{p\left(C=c_{2}\right)\times p\left(T\geq t|C=c_{2}\right)}{p\left(T\geq t\right)}$$

The threshold is the score that maximizes the log ratio of the class posteriors as in equation 5.

(5) $$\underset{t}{\arg\max}\left(\log\left(p\left(C=c_{1}|T\geq t\right)\right)-\log\left(p\left(C=c_{2}|T\geq t\right)\right)\right)$$

Note that *c*_1 _and *c*_2 _represent the mixed and the control classes. The evidence, *p*(*T *≥ *t*), does not need to be calculated. Probabilities are calculated with respect to the candidate set that was determined in the previous step. The goal of the classifier is to remove (*i*) control sequences, and (*ii*) sequences that belong to the mixed set and have scores similar to the scores of control sequences. Given that the scores of controls are due to noise, it is appropriate to assume that they are normally distributed. CrmMiner searches for a threshold starting at the minimum score of controls with increments of 0.1, and ending at one standard deviation above the average score of controls in the candidate set. A threshold within this range can eliminate up to 84.2% of the noise due to the normal distribution properties.

#### Optimizing CrmMiner's parameters

CrmMiner is controlled by six parameters. We designate the validation set to fine tune these parameters. During validation, CrmMiner uses the motif pair list (the regulatory signature) and the threshold that were determined at the training stage. The goal is to find a combination of the six parameters that enables CrmMiner to perform successfully on both of the training and validation sets. CrmMiner's performance is considered satisfactory if the predicted CRMs are significantly more enriched in the mixed set than in the control set. We use the one-tailed Fisher's exact test to obtain a P-value as a measure of statistical significance. The results are considered successful if the E-value*_h _*> 1 and the P-value is less than a threshold. Since CrmMiner performs multiple tests on the training and the validation sets, we use the Bonferroni correction to decide the significance threshold (0.05/the number of tests). For example, we searched 1080 parameter combinations in our experiments described in the next section. Hence, the significance threshold is 4.6*e*^−5^(0.05/1080). In this study, sequences in the training mixed set were gathered from 80 gene loci. Therefore, we tried CrmMiner with combinations of the following six parameters:

• Distance between two motifs defining a pair: {25, 50, 100, 150, 200, ∞}.

• Minimum number of pair occurrences in the mixed set: {7, 8}.

• Maximum pair enrichment value, E-value*_p_*: average + {2, 2.5, 3, 3.5, 4} standard deviations.

• Minimum set enrichment value, E-value*_h_*: {3, 5}.

• Step size of the greedy algorithm, *k*: {1, 5, 10}.

• Maximum number of candidate sequences collected from the mixed set: {160, 240, 320}.

One of two criteria can be used to determine the best parameter combination. First, the best parameter combination is the one that results in the highest significant E-value*_h _*on the validation set. Alternatively, we may search for a combination that results in the most significant validation P-value. For either criterion, we require a significant P-value and E-value*_h _*> 1 during training.

#### Testing

We use the training set to obtain the regulatory signature and a threshold to predict CRMs. CrmMiner parameters are optimized on the validation set. Finally, the performance of the optimized system is assessed blindly on the testing set. A testing E-value*_h _*> 1 and a P-value < 0.05 suggest that CrmMiner found a common regulatory signature across the three sets.

Next, we illustrate the procedures used to construct the mixed and control sets. The majority of the mixed sets in our study were assembled from conserved non-coding elements (CNEs). The control sequences were sampled from the non-coding regions of the human genome.

### Data

In this section, we give details of collecting the data sets. CrmMiner requires a mixed set and a control set. The mixed sets were gathered from CNEs located in tissue-specific gene loci, and the control sets were collected from non-coding genomic regions. In the following, we discuss: (*i*) how the tissue-specific genes were selected, (*ii*) how gene loci were determined, (*iii*) how the CNEs were processed, and (*iv*) how we obtained the control sequences.

#### Determining cell-type-specific and tissue-specific genes

Gene expression data in the GNF Novartis Atlas 2 [36] are a valuable resource for our study. A tissue-specific gene is expected to be expressed in a certain tissue more than other tissues. To select a group of tissue-specific genes, a gene expression level is ranked relative to its expression levels in other tissues. Specifically, we calculate the z-score of a gene expression level with respect to its expression levels in 72 non-cancerous tissues (Equation 6).

(6) $$s_{g}=\frac{e_{g}-mean_{g}}{std_{g}}$$

*s_g _*is the z-score of gene *g*, *e_g _*is the expression level, *mean_g _*is the average expression of *g *in all tissues, and *std_g _*is the standard deviation of *g*'s expressions in all tissues. We ranked genes according to *s_g _*in a descending order. Finally, the top *n *genes are selected.

#### Determining gene loci

We determine the genomic location of genes using the hg18 RefSeq annotation [37]. The boundaries of a gene locus are the midpoints between the gene and each of the two flanking genes. If the gene is the first or last known gene on the chromosome, the locus starts or ends 100 kbp away from the start or the end of the gene of interest.

#### Processing CNEs

We obtained CNEs from the ECR browser [38]. CNEs are human sequences, hg18, that are at least 70% identical with the mouse genome, mm9. If a CNE included a coding sequence, we removed the coding part and kept the rest of the CNE. The shortest CNE in our study is 100 bp long. CNEs including less than 50% repeats were considered. CNEs in the vicinity of tissue-specific genes comprise a mixed set.

#### Collecting genomic control sequences

Our database of control sequences contains 70960 random sequences extracted from non-coding regions of the human genome. The control sequences are similar to CNEs in length, GC content (within 1%), and repeats content (within 1%) [9]. In this study, we constructed a control set specific to a mixed set. The distribution of sequence length in the control set is similar to that of the mixed set. We matched sequences in the mixed set to controls in the database according to ranges of 100 bp. For example, sequences of length 100-199, 200-299, ... , and 1900-1999 bp are matched with control sequences of length in the same ranges. If the mixed set includes sequences longer than 2000 bp, these sequences are matched with control sequences longer than 2000 bp. The ratio of sequence number in the mixed set to that in the control set is 1 to 5.

In the next section, we evaluate CrmMiner on several data sets. First, CrmMiner performance is evaluated under controlled settings. Then, we evaluate CrmMiner on real data sets.

## Results

Our efforts resulted in a software called CrmMiner. The software is available as additional file 1. CrmMiner has two specific goals. First, CrmMiner mines the vicinity of related genes for tissue-specific CRMs. In addition, CrmMiner searches for a common regulatory signature describing the predicted CRMs. This signature consists of pairs of motifs, which represent known vertebrate transcription factor binding sites. We start by defining the evaluation measures. Then, we report the following: (*i*) the performance of CrmMiner under controlled settings, (*ii*) a comparison between CrmMiner putative CRMs and experimentally validated CRMs, (*iii*) the application of CrmMiner to predict CRMs specific to 72 human tissues, and (*iv*) the application of CrmMiner to a group of genes functionally related to the human heart development.

To assess the performance of CrmMiner, evaluation criteria had to be determined. We used a statistical measure, E-value*_h _*(Equation 2), to evaluate the performance of CrmMiner. Recall that the E-value*_h _*quantifies the enrichment of the predictions with sequences from the mixed set. To determine the significance of the enrichment, the Fisher's exact test was applied to obtain a P-value. In addition, when experimentally validated CRMs are available, CrmMiner is evaluated in terms of the true positive (TP), false positive (FP), false negative (FN), and true negative (TN) predictions. When applicable, we used the sensitivity (Equation 7), the specificity (Equation 8), and the precision (Equation 9) to evaluate the performance of CrmMiner.

(7) $$\text{Sensitivity}=\frac{TP}{TP+FN}$$

(8) $$\text{Specificity}=\frac{TN}{TN+FP}$$

(9) $$\mathrm{Pr}\text{ecision}=\frac{TP}{TP+FP}$$

### Controlled Experiments

#### Mining for CRMs specific to the human CD4^+ ^T cells under controlled settings

The vicinity of related genes include CRMs and other genomic sequences. To mimic this setting, we designed special mixed sets consisting of the human CD4^+ ^T cells's DNase I Hypersensitive Sites (HSSs) [2] and background sequences. HSSs mark DNA elements that take part in gene transcription. HSSs often include transcription factor binding sites. Also, they are strong indicators of CRMs. The exact number of CRMs among HSSs is unknown; however, HSSs are enriched with CRMs. The goals of this experiment are (*i*) to study the effect of the number of background sequences in the mixed set on CrmMiner performance, and (*ii*) compare the performance of CrmMiner to that of CisModule [19,39], a related CRM predictor.

To begin, the loci of the top 150 genes, which are mainly expressed in the human CD4^+ ^T cells, were determined. Seventy-five loci were used for constructing the training mixed sets; 37 and 38 loci were used to construct the validation and testing sets. The training, validation, and testing loci included 740, 445, and 443 HSSs, respectively. We considered HSSs that are at least 100 bp long and include at most 50% repeats. We assembled six groups of mixed sets. The training and validation mixed sets included the respective HSSs in addition to *x *× *l *background sequences, *x *= 0, 5, 10, 15, 20, 25 and *l *is the number of loci used to assemble the set. The testing mixed set contained HSSs only. The control sets included three times as many sequences as their mixed sets counterparts. We controlled for sequence length while selecting control sequences (see Methods).

The underlying principle of a related CRM predictor, CisModule [19], is that TFBSs usually cluster within CRMs. CisModule is based on a Bayesian hierarchical model to learn overrepresented motifs that are "co-localized" in CRMs. The algorithm is unique in its ability to learn CRMs while it is learning the over-represented motifs. Both CisModule and CrmMiner can be applied to mine for CRMs in the vicinity of tissue-specific genes. We trained CrmMiner on the training sets and optimized the parameters on the validation sets. The performance of CrmMiner was evaluated on the testing sets. To compare, we followed the same procedure to obtain CisModule predictions. Specifically, we trained CisModule on the training set to find regulatory modules consisting of 3-5 motifs. The number of motifs that resulted in the best performance on the validation sets was used to obtain predictions from the testing sets. Both CrmMiner and CisModule succeeded on the six testing sets. In other words, the testing E-value*_h _*was more than 1 and the P-value is less than 0.05 (Fisher's exact test).

**Figure 2:**
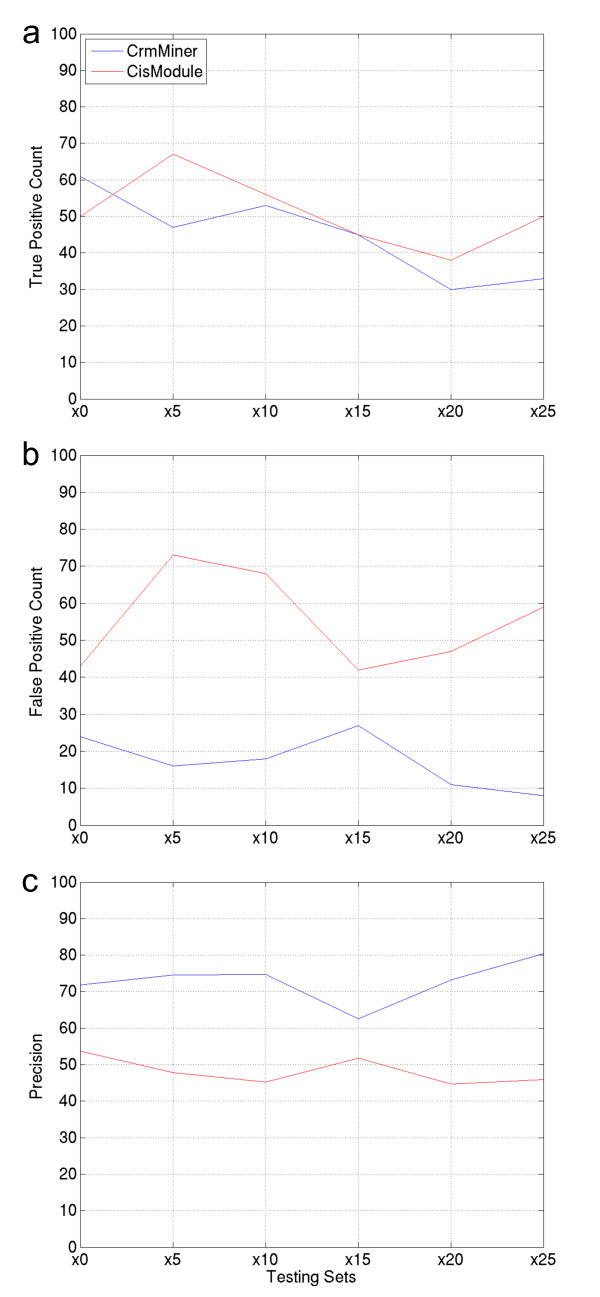
**Performances of CrmMiner and CisModule on controlled data sets**. The performance is measured in terms of the true positive count, the false positive count, and the precision (Equation 9). A set name indicates the number of folds of background sequences mixed with the training and validation hypersensitive sites (HSSs). For instance, the training mixed set of "x10" included 740 HSSs located in the vicinity of 75 genes specific to the human CD4^+ ^T cells. In addition to the 740 HSSs, this training mixed set contained 750 (10 × 75) background sequences. All testing mixed sets consisted of 443 HSSs i.e. they were not mixed with background sequences. All testing control sets were composed of 1125 random genomic sequences. The exact number of CRMs among the HSSs is unknown; however, HSSs are enriched with CRMs.

The true positive rates, the false positive rates and the precisions (Equation 9) of both methods were analysed. We considered a putative CRM as a true positive if it overlapped with a HSS. As expected, CrmMiner's true positive rate declined as the number of background sequences in the training mixed set increased (Figure 2(a)). CrmMiner true positive predictions were more than those of CisModule on the initial set (*x *= 0). The true positive rates of both methods were comparable when *x *= 10 or *x *= 15. CisModule true positive rates were higher than those of CrmMiner when *x *= 5, *x *= 20, or *x *= 25. Recall that the exact number of CRMs among HSSs is unknown. Therefore, we were not able to determine the sensitivities (Equation 7). To count the false positives, positive control predictions that overlapped with HSSs were removed. In the six tests, CrmMiner's false positive rates were lower than those of CisModule (Figure 2(b)). Finally, we calculated the precisions of both methods. We found that CrmMiner was 21% (*x *= 15) - 75% (*x *= 25) more precise than CisModule (Figure 2(c)). These results show that CrmMiner can reliably mine for CRMs in noisy data sets demonstrating its ability to retrieve functional CRMs.

#### Results on random sets

The purpose of this experiment is to ensure that the observed enrichments were not due to a property of the algorithm, or of the predicted TFBSs. We assembled 72 data sets in which the mixed sets were sampled from random genomic sequences. Sequences of the mixed sets, i.e. the pseudo-mixed sets, and the control sets were random genomic sequences. The pseudo-mixed sets and the control sets were similar in sequence length distribution. A control set included five times as many sequences as its pseudo-mixed set. The pseudo-mixed sets were unlikely to share the same set of enriched motif pairs. When CrmMiner was evaluated on the 72 testing pseudo-mixed sets, CrmMiner failed, as expected, to predict the regulatory signature for all of them. These results suggest that the CRMs were predicted due to the shared regulatory signatures and not due to a property of the algorithm or the motifs representing the TFBSs.

### Experiments

#### Mining for CRMs specific to the human heart

In this experiment, we applied CrmMiner to search for conserved CRMs in the vicinity of heart-specific genes. Specifically, we searched for CRMs among the human-mouse CNEs. The goal of this experiment is to apply CrmMiner to retrieve known conserved CRMs by analysing CNEs located in the vicinity of heart-specific genes.

We obtained 160 heart-specific genes (see Methods). The loci of 154 of these genes were determined; the other 6 genes were located in random fragments of the genome. The 154 loci were randomly divided into 75 loci for training, 38 loci for validation, and 39 loci of testing. We assembled the three mixed sets (training, validation, and testing) from the human-mouse conserved non-coding elements (CNEs). Initially, the mixed sets included CNEs located between 10 kbp upstream and 10 kbp downstream of transcription start sites (TSSs). We label this region, 10TSS10. Then, we assembled additional eight sets by expanding the initial mixed sets to include the closest *n *CNEs to TSSs, if they were not already included. We assigned *n *to 0 (CNEs in the 10TSS10 regions only), 10, 20, 30, 40, 60, 80, 100, and ∞ (all). About 75% (116/154) of the loci included 30 or less CNEs. Therefore, when *n *= 30, the mixed sets included all CNEs of at least 75% of the loci. The control sets had five times as many sequences as their mixed sets. Both the mixed and control sets have similar distribution of sequence lengths. Table 1 gives the details of the nine data sets.

**Table 1:** Mining for heart-specific CRMs - input data

n	CNEs/Locus	CNEs	Controls
0	9.6	1480	7400
10	10.8	1667	8335
20	13.1	2019	10095
30	15.3	2358	11790
40	17.5	2692	13460
60	20.9	3219	16095
80	23.7	3644	18220
100	25.7	3959	19795
All	31.9	4911	24555

The mixed sets consisted of CNEs found in the vicinity of 154 heart-specific genes. Nine sets were constructed. All mixed sets contained CNEs within 10 kbp upstream and 10 kbp downstream of the transcription start sites (TSSs). We expanded the mixed sets by including *n *CNEs that are closest to the TSSs, if they were not already included, *n *= 0, 10, 20, 30, 40, 60, 80, 100, ∞. A control set included five times as many sequences as its mixed set counterpart. We controlled for sequence length while assembling the control sets.

CrmMiner was trained on the training sets. For each of the nine sets, we trained CrmMiner and optimized the parameters on the training and the validation sets (we used 288 parameter combinations instead of the 1080 combinations mentioned in the Methods section). The enrichment of the predicted CRMs with CNEs, E-value*_h_*, was calculated. The Fisher's exact test was used to obtain a P-value to determine the significance of the enrichment. We selected the parameters that result in the most significant P-value on the validation set. Both of the training and validation P-values were required to be less than 1.7*e*^−4^(0.05/288), due to the Bonferroni correction. We evaluated CrmMiner, with the best parameters, on the testing sets. Interestingly, CrmMiner succeeded in the nine tests. CrmMiner predictions (additional file 2) were significantly more enriched (3.5-11.2 fold) with CNEs than control sequences (Table 2).

**Table 2:** CrmMiner's performance on the 154 heart-specific genes

n	CNEs	Controls	PCRMs	Controls(+)	E-value*_h_*	P-value
0	375	1875	41	30	6.8	1.4*e*^−15^
10	492	2460	46	55	4.2	4.3*e*^−12^
20	537	2685	19	8	11.2	7.3*e*^−10^
30	680	3400	50	47	5.3	1.3*e*^−15^
40	602	3010	41	45	4.6	1.3*e*^−11^
60	768	3840	40	58	3.5	8.4*e*^−9^
80	656	3280	38	43	4.5	1.4*e*^−10^
100	1107	5535	32	32	5.0	6.5*e*^−10^
All	892	4460	14	15	4.7	7.6*e*^−5^

CrmMiner was evaluated on nine testing sets that were composed of CNEs found in 39 heart-specific gene loci. PCRMs stand for putative CRMs. Controls(+) stand for CRMs predicted among the control sequences. All mixed sets included CNEs in the region between -10 kbp and +10 kbp. Mixed sets were expanded as before. The number of CNEs in the mixed set did not always increase when the number of the closest CNEs, *n*, was increased because we randomly partitioned the loci into three sets every time *n *was increased. The Fisher's exact test was used to calculate the P-values.

We previously developed a supervised-learning method to define the heart regulatory code from 77 known heart enhancers. We used this code to mine the sequence of the human genome and predicted about 42000 putative heart enhancers (PHEs) [9]. To compare CrmMiner predictions to the PHEs, we started with scrutinizing the predictions obtained from the first set (*n *= 0). This set consisted of 1480 CNEs, 91 of which overlapped with the PHEs. CrmMiner predicted 214 CRMs, 50 of which overlapped with the PHEs (expected overlap: 13.1). Our predicted CRMs were 3.8-fold enriched with the PHEs (Z-score = 11.4, P-value = 0). The expected overlap between the two sets was calculated by sampling 10000 random sets from the 1480 CNEs. Each of the 10000 random sets included 214 CNEs. We averaged the results of the 10000 trails, and obtained the P-value by converting the Z-score, which is based on the two-sets overlap distribution of the 10000 trials. As we expanded the mixed set, the predicted CRMs continued to significantly overlap with the PHEs (Table 3) up to 7.5 folds. These results show the agreement between the two methods, even though CrmMiner was not trained on any known heart enhancers.

**Table 3:** Comparison between CrmMiner predictions and putative heart enhancers (PHEs)

n	CNEs	PCRMs	Expected	Enrichment (fold)	Z-scores
0	91	50	13.1	3.8	11.4
10	96	56	13.9	4.0	12.6
20	108	58	6.4	7.5	21.7
30	118	69	9.9	7.0	20.1
40	129	64	11.1	5.8	17.1
60	150	63	10.4	6.1	17.3
80	164	79	10.6	7.5	22.2
100	177	48	6.7	7.2	16.7
All	205	28	5.9	4.7	9.4

The PHEs were obtained by a supervised-learning method [9]. Columns CNEs and PCRMs display the number of overlaps between the CNEs and PHEs, and the PCRMs (putative CRMs) and PHEs, respectively. The expected number of overlaps between the CNEs and the PHEs was calculated experimentally in 10000 trials. In each trial, a set was randomly selected from the input CNEs. These CNEs are located in the 154 heart-specific gene loci and comprise the mixed sets that CrmMiner analysed. The number of CNEs in a random set is the same as the number of the PCRMs. Then, the overlaps between a random set and the PHEs were counted. The expected number is the average overlap in the 10000 trials. The Z-scores were based on the distribution of the overlaps in the 10000 trials. The P-values associated with the nine Z-scores are 0.

Finally, we searched for further evidence to show that several of CrmMiner predictions are functional CRMs in the human heart. We previously collected a set of 95 experimentally validated human heart enhancers [9]. We assessed CrmMiner sensitivity (Equation 7) to detect known heart enhancers present in the mixed set. As a starting point, CrmMiner sensitivity was evaluated on the first set, *n *= 0. This set consisted of 7400 control sequences and 1480 CNEs, which are located in the 10TSS10 regions. The mixed set included 12 known heart enhancers. CrmMiner predicted 214 CRMs including 10 out of the 12 heart enhancers (83% sensitivity). One may argue that these results are due to the abundance of enhancers in the 10TSS10 regions. Hence, these results might be obtained by selecting any 214 CNEs of the 1480 input CNEs. To disprove this assumption, we calculated the expected overlap between the 12 heart enhancers and any 214 CNEs from the mixed sets, as before. We found that 1.7 enhancers are expected to overlap with a set of 214 CNEs. CrmMiner predictions overlapped with heart enhancers about six-fold higher than the expected overlap (Z-score = 6.7, P-value = 1.1*e*^−11^). We obtained the P-value by converting the Z-score. Further, when the number of sequences in the mixed set was increased, CrmMiner remained sensitive to heart enhancers (Table 4). For example, CrmMiner detected 11 out of 15 enhancers present in the seventh set (*n *= 80, sensitivity = 73%, expected overlap = 1, P-value = 0). This set included 3644 CNEs and 18220 control sequences. These results demonstrate CrmMiner ability to locate functional heart enhancers.

**Table 4:** Comparison between predicted CRMs and experimentally validated heart enhancers

n	CNEs	PCRMs	Sensitivity	Expected	P-value
0	12	10	83%	1.7	1.1*e*^−11^
10	13	9	69%	1.9	8.0*e*^−09^
20	15	10	67%	0.9	0.0
30	15	11	73%	1.3	0.0
40	15	8	53%	1.3	1.9*e*^−10^
60	15	9	60%	1.0	4.4*e*^−16^
80	15	11	73%	1.0	0.0
100	16	9	56%	0.6	0.0
All	16	5	31%	0.5	1.0*e*^−11^

We calculated the overlaps of the CNEs found in the 154 heart-specific loci and the putative CRMs (PCRMs) with 95 experimentally validated heart enhancers [9]. Columns CNEs and PCRMs display the number of overlaps between the CNEs and heart enhancers, and the PCRMs and heart enhancer, respectively. The expected number is the average overlap between a random CNE set and the heart enhancers. We selected 10000 random sets as before. The P-values were based on the Z-scores calculated from the distribution of the overlaps in the 10000 trials.

#### Mining for CRMs specific to the human CD4^+ ^T cells

We took advantage of experimental data to evaluate CrmMiner performance on the human CD4^+ ^T cells. Recently, DNase I hypersensitive sites (HSSs) in the same cell type have been determined experimentally [2]. HSSs are indicators of regulatory elements. We used CrmMiner to predict CRMs modulating 160 genes specific to the human CD4^+ ^T cells. We started with CNEs located in the 10TSS10 regions. Then we expanded this set by adding the closest *n *CENs to TSSs. CrmMiner predictions were statistically significant up to *n *= 20 (testing E-value*_h _*= 2.58, P-value = 0.0026, Fisher's exact test). These predictions are provided as additional file 2 (we used 288 parameter combinations instead of the 1080 combinations mentioned in the Methods section).

The initial set (*n *= 0) consisted of 1531 CNEs and 7655 control sequences. Predictions made at the training, validation, and testing stages were combined. In total, CrmMiner predicted 148 CRMs from the mixed set. In addition, 134 control sequences were predicted as positives. Since the exact number of CRMs among HSSs is unknown, the sensitivity cannot be determined. We used the precision and the specificity measures to evaluate the performance of CrmMiner. The 148 potential CRMs overlapped with 121 HSSs (Precision = 82%, i.e 8 in every 10 predictions are true positives). In comparison to 10000 random simulations, we found about 60 of 148 input CNEs are expected to overlap with HSSs. Therefore, the average precision of the random simulations is 41%. CrmMiner precision is two-fold higher than the average precision of the random simulations. When the 134 positive controls were examined, 53 of them were found to overlap with HSSs (Precision = 40%). The average precision obtained from the 10000 random simulations on the control set was 4.5%. CrmMiner precision on the control set was about nine-fold higher than the average precision of the random simulations. CrmMiner achieved specificity of 99%. Recall that the control sequences are random genomic sequences that are similar to the tissue-specific CNEs in length only. Therefore, the precision on the control set and the specificity give estimates of the precision and the specificity of CrmMiner on the human genome.

The results on the enlarged set, *n *= 10, were similar (data not shown). The following are the results on the largest set, *n *= 20. The precision on the mixed set was 67% (average precision of the 10000 random simulations: 31%). The precision on the control set was 33% (average precision of the 10000 random simulations: 5%). CrmMiner specificity was 99%. These results show that many of CrmMiner predictions are functional regulatory elements specific to the human CD4^+ ^T cells.

#### Mining for CRMs specific to 72 human tissues and cell types

Gene expression data of 72 non-cancerous human tissues are currently available [36]. CrmMiner was applied to the top 160 tissue-specific genes, divided into training (50%), validation (25%), and testing (25%) sets. For each tissue, we assembled the mixed sets from the CNEs located in the vicinity of the 160 genes. The control sets were assembled as before (see Methods). Initially, we applied CrmMiner to mixed sets that consisted of the CNEs located in the 10TSS10 regions (*n *= 0). Then the mixed sets were expanded by increasing *n *to 10, 20, 40 and 80. Because experimentally validated CRMs are not available for the majority of tissues, we used the E-value*_h _*to evaluate the performance. Recall that the E-value*_h _*measures the enrichment of CrmMiner predictions with CNEs in contrast to control sequences. CrmMiner performance on a testing set was considered successful if the E-value*_h _*is greater than 1 and the enrichment with CNEs is significant (P-value < 0.05, Fisher's exact test). We evaluated CrmMiner on the 72 testing sets. CrmMiner predictions for the 72 tissues/cell types are provided as additional file 3. CrmMiner succeeded in 85% (61/72) of the initial sets (*n *= 0). When *n *was increased to 10, 20, 40, and 80, CrmMiner succeeded in 82% (59/72), 79% (57/72), 60% (41/72), and 36% (26/72) of the tissues, respectively. Table 5 lists the performance of CrmMiner on the testing sets (*n *= 20) of the 57 tissues.

**Table 5:** CrmMiner performance on 57 human tissues and cell types

Tissue	E-value*_h _*	P-value	Pairs	The Three Most Enriched Pairs
subthalamic nucleus	6.72	2.1*e *^−10^	78	E2F1-TFIII E2F1DP2-ZBED6 GAF-NFMUE1
prefrontal cortex	6.60	5.7*e *^−15^	58	SP1-PITX3 PAX4-BEN EFC-E2A
cingulate cortex	6.44	4.3*e *^−13^	37	MAZR-NFAT2 PAX9-IK3 HEB-AHRARNT
heart	5.52	4.4*e *^−20^	89	PAX9-PXRRXR RSRFC4-E47 CETS1P54-NRSE
caudate nucleus	5.27	5.6*e *^−14^	20	SP1SP3-SP1 SP1-ETF MZF1-TAXCREB
prostate	5.03	4.2*e *^−8^	79	E2F-UF1H3BETA NFY-AP2GAMMA MSX3-SP3
amygdala	4.61	7.3*e *^−10^	34	ZFP281-RFX AHR-CKROX ETF-SP4
BM-CD71^+ ^early erythroid	4.56	1.5*e *^−8^	90	PAX4-NRF1 NFY-KROX MOVOB-NFAT2
adrenal gland	4.40	5.7*e *^−7^	71	STAT6-ZF5 SP1-GATA3 NKX11-SP4
fetal brain	4.26	5.2*e *^−15^	90	VDR-E2F1 OBOX2-PPARA BEN-ETF
bone marrow	4.16	1.8*e *^−7^	68	CDXA-SP1 NFKB-E2F1 AR-PR
BM CD34^+^	4.15	6.0*e *^−9^	130	NRSE-E2F1 E2F1-SP1 E2F-SP1
fetal thyroid	4.13	5.3*e *^−8^	108	E2F-SP4 NFY-ZF5 STAT3-MEF2
bronchial epithelial cells	4.09	7.4*e *^−7^	85	HOXD3-CEBP KLF15-CEBP NKX25-HEN1
occipital lobe	4.09	6.4*e *^−4^	38	EGR2-DEAF1 AHR-CACCCBINDINGFACTOR E2F1-SREBP
whole brain	4.05	3.5*e *^−12^	21	MAZ-ETF SP1SP3-GKLF MZF1-CTCF
PB-BDCA4^+ ^dentritic cells	4.04	2.3*e *^−9^	98	ATF1-ETF AP1-PEBP AP2-PPARA 1
placenta	3.82	1.6*e *^−11^	96	COMP1-GKLF GKLF-NFY CTCF-AP1
hypothalamus	3.82	5.3*e *^−5^	80	GADP-ERR1 AP1-FOXO1 OCT-PAX5
liver	3.73	5.7*e *^−9^	97	COUP-SZF11 COUPTF-SRF GATA4-GFI1
thyroid	3.65	8.6*e *^−8^	93	MTATA-OLF1 MUSCLE-CHX10 LBX2-SP4
thymus	3.65	5.0*e *^−7^	93	IK1-KLF15 MINI19-ALPHACP1 IK1-GKLF
spinal cord	3.64	1.5*e *^−6^	68	IRF-LRF REX1-GLI3 ALPHACP1-PPARA
uterus	3.58	2.9*e *^−8^	85	ELK1-UF1H3BETA TAL1-E2F1 AP2ALPHA-PITX1
pons	3.57	6.1*e *^−5^	108	IPF1-CART1 NMYC-CNOT3 PAX4-MYOGNF1
trachea	3.48	1.1*e *^−9^	110	TRF1-TFIII DEC-CP2 COUPTF-STAT4
lung	3.47	3.0*e *^−7^	36	AP2-TR4 MAZ-CBF SP1SP3-AP1
tonsil	3.42	0.0019	60	HEN1-NFKAPPAB65 PAX4-SMAD E2F6-NRSF
PB-CD19^+ ^Bcells	3.40	9.5*e *^−8^	61	ESE1-PAX6 PAX4-TBX5 MZF1-NRF2
721 B lymphoblasts	3.35	1.8*e *^−6^	104	SP1SP3-E2F4DP1 STAT4-NRF2 ZIC1-EGR1
testis	3.30	4.8*e *^−4^	73	AP2-ATF T3R-HNF4 MYOD-SRF
uterus corpus	3.27	1.3*e *^−8^	91	TCF4-POU6F1 CAAT-PITX3 PAX4-XVENT1
tongue	3.24	3.8*e *^−7^	72	ZFP281-NFY SP3-PITX3 AP2-IK2
temporal lobe	3.21	3.7*e *^−7^	95	TBX5-MTF1 MTATA-HIC1 ERG-ZIC3
PB-CD56^+ ^NKCells	3.12	3.1*e *^−5^	53	NFY-SP1 CMYB-STAT6 SRF-SP4
smooth muscle	3.11	2.9*e *^−6^	87	KROX-MEF2 SP1-XBP1 HIF1-IK
pituitary gland	3.06	2.2*e *^−6^	96	MTATA-MAZR ZF5-WT1 AHR-ETF
adrenal cortex	3.04	1.5*e *^−5^	111	MYCMAX-PAX4 SP2-E2F1 VJUN-ZNF219
BM-CD105^+ ^endothelial	2.95	0.0049	79	E2F-SMAD4 AP4-PAX4 P53-NFY
lymph node	2.94	2.6*e *^−5^	131	CETS1P54-GABP HIF1-NRSE PEA3-MTF1
adipocyte	2.91	1.3*e *^−6^	79	AP2-NFY E2F1-LXR CNOT3-IK2
skeletal muscle	2.89	7.6*e *^−7^	80	P53-CACD MEIS2-STAT1 MYOD-XVENT1
thalamus	2.86	0.0065	42	PLAG1-PAX3 AP1-CMAF MZF1-GATA2
pancreatic islets	2.79	6.7*e *^−8^	97	CDXA-PROP1 MEIS1-ZBED6 NFKB-E2F
skin	2.79	7.8*e *^−5^	73	CACBINDINGPROTEIN-XVENT1 NFKB-ZTA ER-SREBP1
medulla oblongata	2.78	7.9*e *^−7^	103	ZNF515-NKX25 CETS1P54-NKX26 P53-CMYB
whole blood	2.77	5.8*e *^−5^	53	PU1-SP2 GFI1-CTCF AP2-GR
cardiac myocytes	2.69	9.1*e *^−4^	120	EGR-MECP2 SRY-CNOT3 E2F-EGR3
fetal lung	2.62	1.7*e *^−4^	55	HIF1-LMAF CTCF-MECP2 AML2-CP2
PB-CD4^+ ^Tcells	2.58	0.0026	121	NGFIC-EGR3 ZFP206-CBF AHRARNT- COUP
cerebellum peduncles	2.52	1.2*e *^−4^	74	ZNF219-NFY KLF15-OBOX2 NKX11-ZNF219
olfactory bulb	2.31	4.3*e *^−4^	70	NFY-SP1 NFKAPPAB-CTCF CP2-GLI
cerebellum	2.30	0.0034	58	AP2-LBX2 COUPTF-E2F E2F-SREBP
kidney	2.29	4.08*e *^−5^	87	GCM-ARP1 E2A-RORBETA NFY-TFIII
BM-CD33^+ ^myeloid	1.80	0.0489	92	SRF-HMGIY PR-STAT6 T3R-AP1
parietal lobe	1.78	0.0443	82	AP2ALPHA-E2F1 HIF1-GABP BEN-MYF
appendix	1.75	0.0285	88	AP1-LXR PAX2-PAX4 CNOT3-CREB

We trained, validated and tested CrmMiner on 72 human tissues and cell types [36]. The mixed sets consisted of CNEs in the vicinity of 160 tissue-specific genes. Those CNEs were located within 10 kbp up or downstream of the TSSs, or were among the closest 20 CNEs to the TSSs. Here, we report CrmMiner performance on the testing sets. The number of motif pairs comprising the tissue-specific signatures are listed under the Pairs column. The P-values were obtained by one-tailed Fisher's exact test.

Next, CrmMiner predictions, obtained from one of the expanded sets (*n *= 20), are analysed. We assessed whether CrmMiner located potential CRMs in all of the tissue-specific gene loci. CrmMiner predicted CRMs located in 57% of the loci. Three reasons can account for loci without putative CRMs:

• We searched only for conserved CRMs; however, CRMs may be weakly or not conserved [5,40],

• The missing predictions might be located outside the search range, and

• The missing CRMs may include unknown TFBSs.

On average, CrmMiner predicted 2.4 CRMs per gene. This is a known phenomenon. Genes regulated by more than one CRM were reported in the literature [41,42]. The average length of a potential CRM is 422 bp which is almost twice as long as the average CNE. Recall that we controlled for sequence length while assembling the control set.

The CNEs were distributed as follows: 11.7% were in the promoters (within 2 kpb upstream of the TSSs), 45.2% were in intronic regions, and 43.1% were intergenic, whereas 28.6%, 29.6%, and 41.8% of the putative CRMs were located in promoter, intronic, and intergenic regions. CrmMiner predictions were 2.4-fold enriched with CNEs located in the promoter regions (P-value = 0, Fisher's exact test). Promoter regions are known to include CRMs [41-44]. Therefore, these results suggest that these predictions are likely functional tissue-specific CRMs. In addition, CrmMiner predictions included distal ones. Specifically, 20.8% of the predicted CRMs were more than 50 kbp away from the TSSs. Consequently, the putative CRMs and the regulatory signatures can assist in understanding the tissue-specific gene regulation.

#### Mining for CRMs specific to the developing human heart

CrmMiner can be applied to a group of functionally-related genes for which no expression data are available. We used CrmMiner to predict CRMs that can potentially regulate genes related to the development of the human heart. The availability of such putative CRMs should extend our understanding of the genetic basis of congenital heart diseases. We predicted CRMs located in the vicinity of 93 genes related to the human heart development (GO:0007507). We obtained the functional annotation through the Cardiovascular Gene Ontology Annotation Initiative (http://www.ebi.ac.uk/GOA/CVI/). The 93 genes were divided into 46 for training, 23 for validation, and 24 genes for testing. CrmMiner was initially applied to the CNEs located in the 10TSS10 regions (*n *= 0). Then, the search scope was extended to include the *n *closest CNEs to the TSSs, if these CNEs were not already included. We assigned n to 10, 20, 40, and 80. The control sets were similar to their corresponding mixed sets in sequence length distribution (see Methods). The ratio of the size of the mixed set to that of the control set is 1:5.

We trained CrmMiner on the training sets, and optimized the parameters on the validation sets. Finally, CrmMiner was tested on the testing sets. Regarding the testing sets, the predictions of CrmMiner were significantly more enriched with CNEs than with control sequences. The highest testing E-value_h _was obtained when n was 20 (E-value*_h _*= 3.6, P-value = 1.1*e*^−11^, Fisher's exact test). Based on the analysis of this set (*n *= 20), the predicted regulatory signature consisted of 132 motif pairs (Table 6). The maximum number of nucleotides separating two motifs is 100 nucleotides (determined during the parameter optimization stage). These motif pairs were selected based on their enrichment values (E-value*_p_*, Equation 1) during the training stage. To predict whether a sequence includes a CRM or not, the sequence is scored according to the predicted regulatory signature. Specifically, a sequence score is the sum of the E-value*_p _*of the signature pairs present in the sequence. Those pairs must meet the distance constraint (100 bp). Finally, a sequence that has a score of 23.8 (determined during the training and the parameter-optimization stages) or above was predicted to be a CRM.

**Table 6:** The predicted regulatory signature of the developing human heart

Pair	E-value*_p_*	Pair	E-value*_p_*	Pair	E-value*_p_*
ZF5 & SZF11	12.5	E2F & HIC1	12.5	IRF1 & HIC1	12.5
SOX2 & ZFX	11.2	GATA1 & ZFX	12.5	TGIF & COREBINDINGFACTOR	11.2
AP2 & GATA	11.2	LTF & E2F1	12.5	FOXJ2 & CREL	11.2
MSX3 & CKROX	11.2	AIRE & LMX1	11.2	EGR & NFY	11.2
DAX1 & LXR	11.2	E2F & MYOD	12.5	MEF2 & AP4	11.2
CETS1P54 & DEAF1	11.2	FPM315 & LMO2COM	12.0	ZFP206 & LUN1	11.2
MAZ & BARX2	11.2	BACH2 & COUP	11.2	ZF5 & CAAT	11.2
ATF1 & ZFX	11.2	ALPHACP1 & RNF96	11.2	LHX8 & IK	11.2
SP1SP3 & RFX1	11.0	SP1 & MSX1	11.2	PEA3 & SMAD4	11.0
MOVOB & ELK1	11.0	ZNF219 & GATA2	10.8	ETS & GABP	10.7
STAT1 & RNF96	10.0	PXRRXR & SP1	10.0	XPF1 & EGR1	10.0
POU6F1 & AP2GAMMA	10.0	GATA6 & KROX	10.0	AP2ALPHA & LMX1	10.0
RXRG & RNF96	10.0	GATA1 & AP2	10.0	NFY & CNOT3	10.0
ZIC2 & CMYB	10.0	HIC1 & FOX	10.0	FPM315 & ATF	10.0
EBF & CNOT3	10.0	LMX1 & NKX12	10.0	LEF1 & WT1	10.0
LIM1 & LHX61	10.0	SP4 & GATA	10.0	NFKB & PAX4	10.0
AR & PAX4	10.0	AP2 & AHRARNT	10.0	CNOT3 & LUN1	10.0
ZNF515 & BEN	10.0	NF1 & CAAT	10.0	CNOT3 & ARNT	10.0
AREB6 & RNF96	10.0	AP2ALPHA & NMYC	10.0	NFKB & NGFIC	10.0
MAZR & LMO2COM	10.0	TAL1BETAITF2 & ZIC1	10.0	MYOGNF1 & PAX5	10.0
HEB & EN2	10.0	MINI19 & LIM1	10.0	CPHX & VDR	10.0
SPZ1 & GATA3	10.0	EBF & E2F1	10.0	MEF2 & E2A	10.0
GLI3 & FOXO4	10.0	NFKB & MEIS1AHOXA9	10.0	MSX2 & UF1H3BETA	10.0
ETF & TBX22	10.0	ZBED6 & MEF2	10.0	CNOT3 & PXRRXR	10.0
KROX & NFY	10.0	SP2 & GATA2	10.0	MYOD & MEF2	10.0
ROAZ & E2F1	10.0	GATA6 & UF1H3BETA	10.0	SP1 & GATA	10.0
AML2 & KLF15	10.0	ZF5 & TTF1	10.0	MZF1 & MIF1	10.0
MECP2 & GC	10.0	NFY & WT1	9.1	AREB6 & WT1	9.3
ZIC1 & E2F1	9.3	HIC1 & FOX	9.1	ZNF219 & GTF2IRD1	9.1
EGR & GATA2	9.1	CNOT3 & PU1	9.2	MINI19 & AREB6	9.1
GATA & SP1	9.0	MUSCLE & CREBATF	9.0	STAT3 & E2F1	9.0
HIC1 & HFH4	9.0	MUSCLE & MEF2	9.0	TBX15 & SP3	9.0
CTCF & CPHX	9.0	FOXO3 & PAX5	9.0	PAX9 & PAX4	8.7
NFY & SP1	8.7	EGR & GATA2	8.6	SPZ1 & ZF5	8.4
LBP1 & POLY	8.3	PEA3 & MINI19	8.3	CNOT3 & AHRARNT	8.3
SP1 & SOX10	8.3	BARHL1 & CKROX	8.3	AP2 & ATF1	8.3
AP2 & MEF2	8.3	EGR & DEAF1	8.2	COUPTF & ZF5	8.2
SP1 & NFY	8.1	AMEF2 & TBX15	8.0	TAL1BETAITF2 & SP2	8.0
PXRRXR & HIC1	8.0	AP2 & E2F1	8.0	TGIF & MRG2	8.0
RXRG & ZBED6	8.0	E2A & SRF	8.0	SP3 & ELK1	8.0
CEBP & CEBP	8.0	TATA & ERR1	8.0	E2F1 & SP1	8.0
AML2 & GC	8.0	XPF1 & SREBP	8.0	EGR1 & SMAD	8.0
ZIC1 & PPARG	8.0	DBP & E47	8.0	KROX & HMEF2	8.0
CTCF & GATA3	8.0	EAR2 & ETF	8.0	SP1 & PXRRXR	8.0

CrmMiner predicted 132 motif pairs that comprise the regulatory signature of the developing human heart. The score of a sequence is the sum of the enrichment values (E-value*_p_*, Equation 1) of any of the 132 pairs present in the sequence provided that a pair of motifs meets the distance requirement (≤ 100 bp). A sequence that has a score equal to or above 23.8 is predicted to be a CRM specific to the developing heart. The 23.8 is the threshold that was determined at the training and the parameter-optimization stages. The unit of E-value*_p _*is fold.

The proposed signature included several TFs that regulate genes related to heart development. Examples of such TFs are: E2F [45], MEF2 [46], MYOD/bHLH [47], SRF [48], SP1, SMAD4 [49], SP4 [50], WT1 [51], AP1 [52], and AP2 [53]. This evidence indicates that our putative CRMs are likely to regulate genes modulating heart development. In addition, these results show that CrmMiner can provide useful biological insight in the absence of gene expression data.

## Discussion

In this section we compare CrmMiner to a closely related work and analyze the structure of the predicted regulatory signatures. In addition, the impact of the distance between the two co-occurring motifs on the performance is analyzed. Then, we illustrate the applicability of CrmMiner to all sequences, regardless of their conservation degree, and to experimental data. Finally, additional results are presented to show the consistency in CrmMiner performance on enlarged control sets.

### Comparison to related work

CRM-PI [30] is the work most closely related to CrmMiner. Both tools are based on the same biological principles. Specifically, they use enriched motif pairs to mine for CRMs in the vicinity of related genes. Although these two methods are similar in principle, they differ in the following aspects:

• CRM-PI identifies motif pairs in the "conserved regions" of the tissue-specific promoters, whereas Crm-Miner uses sequences, which are in the vicinity of 50% of the tissue-specific genes, to identify enriched motif pairs. These sequences are located in or outside the promoter regions.

• During training, CrmMiner selects a subset of the enriched pairs simultaneously while it is selecting the candidate CRMs. These two steps are independent in the CRM-PI method.

• All "important" pairs contribute to a sequence potential energy, i.e. a sequence score that the CRM-PI calculates. However, a sequence score calculated by CrmMiner depends on a subset of the enriched pairs.

• In the CRM-PI method, the weight associated with a pair is a function of the enrichment and the distance between the two co-occurring motifs. The shorter the distance between the two motifs is, the higher the weight. We apply a 0-1 weighting scheme to the selected enriched pairs. If the distance between the two motifs is less than a threshold, the weight is 1 × E-value*_p_*; otherwise, the weight is zero. Our weighting scheme is inspired by two biological phenomena. First, it was observed that the interaction between two closely-located TFs is preserved when the distance between their binding sites is altered by up to 100 bp [54]. In other words, the two TFs still interact as long as their binding sites were separated by a maximum of 100 bp. By interaction, we mean that the two TFs produce their effects in synergy with each other. Second, the spacing between TFBSs in CRMs is flexible. It was observed that a complex between two TFs still forms if the distance (bp) between their binding sites is increased by an "integral multiple" of 10 bp [54]. These two phenomena led us to adapt the flexible spacing scheme instead of a spacing system that favors TFBSs that are located close to each other.

• CrmMiner requires three data sets. In comparison, CRM-PI requires one data set.

• We apply the optimal Bayesian classifier to determine a threshold above which a sequence is likely to be a CRM. The classifier is trained on sequences that include at least one of the selected enriched pairs. These sequences are collected from the mixed and the control sets. The threshold used in CRM-PI was determined experimentally on 10000 control sequences. The threshold is the potential energy below which 5% of the control sequences are considered putative CRMs.

### Structure of the tissue-specific regulatory signature

To understand the composition of the regulatory signatures, we analyzed the 57 predicted signatures (*n *= 20). We found that 22% of the TFs were components of more than 25% of the signatures, and 78% were parts of less than 25% of the signatures. For example, ubiquitously expressed TFs such as SP1 and AP2 were parts of 95% (54/57) and 88% (50/57) of the predicted signatures. Tissue-specific TFs such as GATA3 and OTX1 were components of 25% (14/57) and 9% (5/57) of the signatures. These results suggest the following: (*i*) the majority of TFs comprising a regulatory signature are tissue specific, and (*ii*) a tissue-specific regulatory signature is a combination of ubiquitously expressed TFs and tissue-specific TFs. Such results confirm a similar recently published observation [29]. In addition, the interaction between tissue-specific and ubiquitously expressed TFs in a CRM of the *Secretin *gene has been previously reported [55].

### Distance between the two co-occurring motifs

The parameter that controls the maximum distance between two co-occurring motifs has a biological significance. To gain the biological meaning of the parameter, we analyzed the impact of this parameter on CrmMiner performance on 57 tissues. The mixed sets of the 57 tissues consisted of the CNEs that are located in the 10TSS10 regions or are among the closest 20 CNEs to the TSSs. These tissues were chosen because there was statistical evidence indicating that CrmMiner succeeded in predicting their tissue-specific CRMs and the regulatory signatures. The performance of CrmMiner was the best on 89% (51/57) of the tissues when the maximum distance was constrained by a threshold. These results are supported by the phenomenon mentioned above, where two TFs function in synergy as long as the distance between their binding sites is less than a threshold.

### Input sequences

CrmMiner can mine for CRMs in conserved elements based on any conservation scheme. In addition, the user may choose to apply CrmMiner to any sequences regardless of their conservation degree. To illustrate, regions in the vicinity of related genes should be divided into shorter sequences of length, for instance, 400-500 bp. CrmMiner can be applied in an incremental manner. A good starting point is to apply CrmMiner to the non-coding sequences within 10 kbp up or downstream of the TSSs. These regions can then be expanded by 5 kbp in both directions until the signal is lost. As a proof of concept, we applied CrmMiner to the 10TSS10 regions of 160 genes specific to human CD4^+ ^T cells. The non-coding sequences of these regions were divided into non-overlapping sequences of 500 bp in length. We trained, optimized the parameters, and tested on the training, validation, and testing sets. The predicted CRMs of the testing set were significantly more enriched with sequences from the mixed set than with controls (E-value*_h _*= 3, P-value = 1.3*e*^−4^, Fisher's exact test). CrmMiner processed the mixed set which included 4540 sequences and predicted 148 CRMs, 93 of them overlapped with HSSs resulting in a precision of 63%, i.e. 63 of every 100 predictions overlapped with HSSs. In comparison, the average precision of 10000 random simulations was 28%.

Further, we have demonstrated that CrmMiner can predict CRMs by processing tissue-specific HSSs. Recall that not all HSSs include CRMs; they are enriched with CRMs. These results demonstrate the usefulness of CrmMiner to analyse experimental data such as HSSs or histone marks.

### Experimental and predicted TFBSs

Currently, new technology such as the ChIP-seq makes it possible to obtain tissue-specific transcription factor binding sites. In the future, we will use experimentally determined TFBSs or a mixture of experimental and predicted TFBSs to detect tissue-specific CRMs.

### Size of the control set

CrmMiner requires a set that includes CRMs mixed with background sequences and another set of control sequences. In this study, the controls were sampled from the non-coding regions of the human genome. The two sets were similar in their sequence-length distributions. CrmMiner identifies the enriched motif pairs and the putative CRMs by contrasting the two sets. The control set needs to be large enough to accurately compute (*i*) the expected number a motif pair is found in the genome and (*ii*) the threshold that separates CRMs from background sequences. Initially, the control set contained five times as many sequences as the mixed set. We applied CrmMiner to these control sets and mixed sets that consisted of CNEs located in the 10TSS10 regions or were among the 20 closest CNEs to the TSSs. CrmMiner was able to predict the regulatory signatures of 57 tissues (median E-value = 3.2 fold). When the ratio of CNEs to controls was increased to 1:10 and 1:15, CrmMiner succeeded in predicting the regulatory signatures of 55 (median E-value = 3.5 fold) and 55 (median E-value = 3.7 fold) tissues, respectively. These results show the consistent performance of CrmMiner on increased numbers of control sequences.

## Conclusions

In sum, we designed and developed a new system called CrmMiner to predict CRMs modulating related genes. CrmMiner contrasts the mixed sequences to random genomic sequences to identify co-occurring motif pairs that are enriched in the mixed set. A subset of the enriched pairs is used to predict tissue-specific CRMs and the regulatory signature. The following lines of evidence support the validity of our method:

• Under controlled settings, CrmMiner was able to find CRMs specific to the CD4^+ ^T cells in noisy data sets up to a 1:25 signal to noise ratio.

• Although CrmMiner does not rely on experimentally determined CRMs, its potential heart-specific CRMs overlapped significantly with heart enhancers predicted by a supervised-learning method. The model obtained by the supervised-learning technique required known heart enhancers for training.

• Several of the predicted heart CRMs overlapped with experimentally validated heart enhancers.

• CrmMiner precision and specificity in detecting CRMs specific to the human CD4^+ ^T cells reached up to 82% and 99%, respectively.

• As a starting point, we searched for CRMs located in the 10TSS10 regions of co-expressed genes in 72 human tissues and cell types. Statistical evidence suggests that CrmMiner succeeded in predicting CRMs specific to 61 human tissues and cell types. When the signal sets were enlarged to include the closest 20 CNEs to the TSSs, about 21% of the predicted CRMs were located more than 50 kbp from their target TSSs.

• CrmMiner was also proven useful in mining for CRMs in the vicinity of genes related to the development of the human heart. Prior published studies support the validity of the predicted TFs our method links to transcription regulation of the developing heart.

Therefore, CrmMiner can play an important role in learning the genomic signature of tissue-specific CRMs.

## Availability

The CrmMiner software is included as an additional file 1. The most updated version is available from the authors upon request.

**Project name: **CrmMiner

**Operating systems: **Unix/Linux

**Programming language: **Java and Perl

**Other requirements: **Java 1.6 or higher and Perl Statistics-Lite module

**License: **The code provided by the authors, National Center for Biotechnology Information (NCBI), National Library of Medicine, is a work of the U.S. Government and is not subject to copyright protection in the United States.

## Abbreviations

CRM: cis-regulatory module; TSS: transcription start site; HSS: DNase I Hypersensitive Site; PHE: putative heart enhancer.

## Authors' contributions

IO and HZG conceived the study. HZG implemented the software. HZG and IO designed the experiments. HZG conducted the experiments and performed the analysis. HZG wrote the manuscript. HZG and IO approve the manuscript.

## Supplementary Material

Additional file 1 **Software**. The CrmMiner software. Click here for file

Additional file 2 **Predictions for three tissues**. This file contains CrmMiner predictions for the human adult heart, developing heart, and the CD4^+ ^T cells. It includes the tissue-specific genes, the predicted signatures, and the putative CRMs. Click here for file

Additional file 3 **Predictions for all tissues**. This file includes predictions for 72 human tissues and cell types. It includes the tissue-specific genes, the predicted signatures, and the putative CRMs. This file also contains predictions for the heart and the CD4^+ ^T cells. We obtained these predictions by searching a larger set of parameters. Click here for file
