# Supplementary material for: Predicting tissue specific cis-regulatory modules in the human genome using pairs of co-occurring motifs
Source: BMC Bioinformatics. 2012 Feb 7;13:25. doi: 10.1186/1471-2105-13-25 (PMC3359238; doi:10.1186/1471-2105-13-25)
Supplement: Additional file 1 — Software. The CrmMiner software. [file 1471-2105-13-25-S1.GZ › crmMiner/README.pdf]

Please contact the author at girgishz@mail.nih.gov or hani.z.girgis@gmail.com if you have any questions regarding installing or running CrmMiner.

Requirements

1] Perl Module Statistics::Lite
  + Can be downloaded from http://search.cpan.org/~brianl/Statistics-Lite-3.2/Lite.pm
  + Install the module.
  + Edit the following line in fully_qualified_path/crmMiner/perl/train.pl: use lib "fully_qualified_path/Statistics/blib/lib";

2] Mast program
  + Can be downloaded from http://meme.sdsc.edu/meme4_6_0/intro.html

3] TRANSFAC Position Weight Matrixes
  + If you have Transfac professional license, download the "matrix.dat" file.
  + Process the matrix.dat file as
    + make a destination directory for the log odds tables
    > java -cp fully_qualified_path/crmMiner/java/bin gov.nih.nlm.ncbi.transfac.ReaderForMatrix fully_qualified_path/matrix.dat fully_qualified_path/destination

Installation

1] Modify CrmMiner so it can find the Java executables, Perl code, MAST, and the log odds tables.
2] Edit the following lines in crmMiner/perl/crmMiner.pl:

```
my $binDir      = ' qualified_path/crmMiner/java/bin' ;
my $perlDir     = ' qualified_path/crmMiner/perl'      ;
my $mastExec = ' qualified_path/mast'      ;
my $pwmDir    = ' fully_qualified_path/logOddsTables';
```

3] There is not need to compile the Java source code.
4] If you wish to compile the Java source code

```
> cd crmMiner
> cd java
> javac -sourcepath src/ -d bin/ src/gov/nih/nlm/ncbi/features/*.java
> javac -sourcepath src/ -d bin/ src/gov/nih/nlm/ncbi/transfac/*.java
> javac -sourcepath src/ -d bin/ src/gov/nih/nlm/ncbi/util/*.java
```

Usage

1] To obtain a list of the parameters and their usage, run crmMiner without arguments:

> full_path/crmMiner/perl/crmMiner.pl

2] The heart fasta files and the predicted transcription factors binding sites are provided.
3] If you obtained a newer version of Transfac, the numbers will be different. We used release 2010.4.
4] Example:

```
> fully_qualified_path/crmMiner/perl/crmMiner.pl fully_qualified_path/crmMiner/test/heart_fasta/inputFiles.txt fully_qualified_path
/crmMiner/test/heart_tfbs 1
```

+ The final line of the output should be:

```
89    5.52    63    57    1.50765521812416    24.6263208896138    4.3978501512403076E-20
```

+ This line represents the performance on the testing set. It can be interpreted as the following:

Number of motifs pairs: 89
E-value: 5.52
Putative CRMs: 63
Control Positives: 57
Maximum log ratio (Equation 5 in the manuscript): 1.50765521812416
Threshold: 24.6263208896138
P-value (Fisher's exact test): 4.3978501512403076E-20

+ If you processed the position weight matrixes, use the same commands as before; change the destination directory and the demo_parameter to 0.

Output

1] Change directory to your destination directory
2] CrmMiner writes the regulatory signature and the putative CRMs to the following files:

selectedPairs.txt: the proposed regulatory signature.
bayes.train.txt: results on the training set.
bayes.query.txt: results on the validation set.
bayes.test.txt: results on the testing set.
train.signal.pstv.coor: putative cis-regulatory modules in the training set.
query.signal.pstv.coor: putative cis-regulatory modules in the validation set.
query.signal.pstv.coor: putative cis-regulatory modules in the testing set.
